# Application of the Cre/*lox*P Site-Specific Recombination System for Gene Transformation in *Aurantiochytrium limacinum*

**DOI:** 10.3390/molecules200610110

**Published:** 2015-06-01

**Authors:** Hengyi Sun, Hao Chen, Xiaonan Zang, Pan Hou, Bingbing Zhou, Yuantao Liu, Fei Wu, Xiaofei Cao, Xuecheng Zhang

**Affiliations:** Key Laboratory of Marine Genetics and Breeding, Ministry of Education, Ocean University of China, Qingdao 266003, China; E-Mails: sunhengyi1987@163.com (H.S.); chenhao4680088@126.com (H.C.); xiaohou_aishengwu@163.com (P.H.); qzwnzn@yeah.net (B.Z.); liuyuantao2418@126.com (Y.L.); wufei0917@sina.cn (F.W.); caoxiao1@126.com (X.C.); xczhang8@163.com (X.Z.)

**Keywords:** antibiotic resistance marker gene, *Aurantiochytrium limacinum*, Cre/*lox*P site-specific recombination system, homologous recombination

## Abstract

The Cre/*lox*P site-specific recombination system was applied to *Aurantiochytrium limacinum* to obtain a transformant without the antibiotic resistance marker gene. First, the enhanced green fluorescent protein gene (*egfp*) and chloramphenicol resistance gene (*Cm^r^*), along with the two *lox*P loci, were integrated into the genome of *A. limacinum* OUC88 using 18S rDNA sequences as the homologous recombination sites. Then plasmid pSH65, containing a zeocin resistance gene (*Ble^r^*) was transferred into *A. limacinum* OUC_CG. After induction with galactose, repeated passage in culture and PCR-based assessment, the pSH65 plasmid was lost and *A. limacinum* OUC_EG host was shown to no longer have resistance to 100 mg chloramphenicol/L or 5 mg zeocin/L. Through southern blotting and fluorescence detection, *egfp* was found to be integrated into the genome of *A. limacinum* OUC_EG, and EGFP was successfully expressed in the cells. The successful application of the Cre/*lox*P system demonstrates an experimental basis for genetic modification of *A. limacinum* so as to obtain transformed strains with no antibiotic resistance marker genes.

## 1. Introduction

In recent years, a genetic transformation method has been successfully established in *Aurantiochytrium limacinum*. A zeocin resistance gene was introduced into *A. limacinum* using particle bombardment [1]. Cheng *et al.* integrated the zeocin resistance gene into the 18S rDNA sequences of *A. limacinum* using electrotransformation and homologous recombination technology [2]. *Agrobacterium tumefaciens*-mediated transformation was also successfully applied to *A. limacinum* and the results demonstrated that the exogenous *egfp* gene had been successfully incorporated into the genome and that it was expressed efficiently [3]. Furthermore, a partial sequence of the *pks* gene in *A. limacinum* was successfully knocked out by electroporation, using the 18S rDNA sequences of *A. limacinum* as homologous recombination sites [4]. However, all these *A. limacinum* transformants retained antibiotic resistance genes, which is adverse for the industrial application of *A. limacinum* and was environmentally undesirable. Thus, research on a new method to eliminate the antibiotic resistance genes in genetic transformations of *A. limacinum* is of significant importance.

The Cre/*lox*P site-specific recombination system was first developed in phage p1 of *Escherichia coli* [5,6] and consists of the Cre recombinase and two *lox*P loci. In the Cre/*lox*P site-specific recombination system, there are two important steps: first, the circular plasmid containing two *lox*P loci are transferred into a host, and then the circular plasmid pSH65 with the Cre recombinase is transferred into the host, where it specifically recognizes the *lox*P sites [7]. Depending on the directivity of the *lox*P site, specific inversion, elimination or translocation of the target fragment is performed by the Cre recombinase. The advantage of the Cre/*lox*P site-specific recombination system is that it can specifically eliminate the antibiotic resistance gene markers that are transformed into the host in the first step, and the pSH65 plasmid can then be lost by repeated sub-culturing of the host after the second step. In 1990, the Cre/*lox*P recombination system was applied successfully in tobacco for site-specific reorganization, which was the first application of the Cre/*lox*P recombination system in plant cells [8]. Subsequently, the Cre/*lox*P recombination system was used to remove the marker genes from genetically modified organisms. This has been performed successfully in yeast and other organisms [9,10,11,12,13]. In this paper, the Cre/*lox*P site-specific recombination system was, for the first time, applied to genetic transformation of *A. limacinum*, where it has provided the experimental basis for directional transformation of *A. limacinum* without leaving behind an antibiotic marker.

## 2. Results and Discussion

### 2.1. Construction of plasmid p18SCPGC

**Figure 1:**
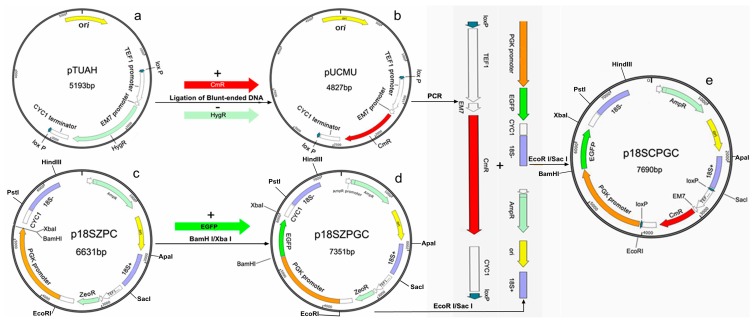
Construction process of the plasmid. (**a**) Plasmid pYUAH; (**b**) Plasmid pUCMU; (**c**) Plasmid p18SZPC; (**d**) Plasmid p18SZPGC; (**e**) Plasmid p18SCPGC.

Plasmid p18SCPGC was successfully constructed using the procedure described in the Experimental section. “0” point was a reference point relative to the location of all genes and sequences (Figure 1e). After sequencing and alignment, it was established that the two homologous 18S rDNA sequences of *A.* *limacinum* were located at 1893–2507 (615 bp of 18S+) and 6605–7284 (680 bp of 18S−), respectively, which constituted the two ends of the transformation fragments. The two *lox*P sequences were located at 2514–2547 and 4017–4050 respectively, and flank the *Cm^r^* antibiotic resistance gene. The coding sequence of the *Cm^r^* gene was at 3032–3691 with the *TEF1* promoter and the *CYC1* terminator at the two ends. The *PGK* promoter sequence was at 4057–5548, which was followed by the coding sequence of the *egfp* gene at 5555–6275. All the sequences mentioned above are consistent with the expectations, which demonstrated that plasmid p18SCPGC was constructed correctly. 

### 2.2. Screening of the Transformant after the First Electrotransformation Step

**Figure 2:**
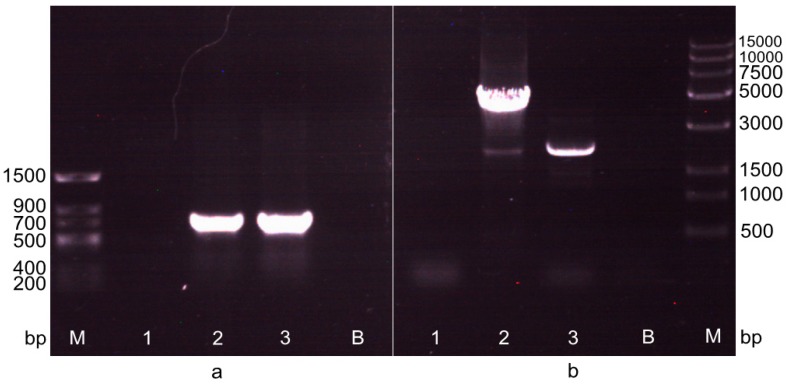
The detection of recombinant in the two transformations by PCR amplification. (**a**) The detection of recombinant in the first transformation by PCR amplification with primers Cm-F/R. M: *Trans* DNA Marker II. 1: Negative control, the PCR amplification band of *Cm^r^* gene with the genomic DNA of *A. limacinum* OUC88 as the template. 2: The PCR amplification band of *Cm^r^* gene with the genomic DNA of *A. limacinum* OUC_CG as the template. 3: Positive control, the PCR amplification band of *Cm^r^* gene with the plasmid pACYCDuet-1 as the template. B: Blank control; (**b**) The detection of recombinant in the second transformation by PCR amplification with primers 18S+-F/P*pgk*-R. M: *Trans*15K DNA Marker. 1: The PCR amplification band with genomic DNA of *A. limacinum* OUC88 as the template; 2: The PCR amplification band (3655 bp) of 18S+-*lox*P-*Cm^r^*-*lox*P-P*pgk* with genomic DNA of *A. limacinum* OUC_CG as the template; 3: The PCR amplification band (2185 bp) of 18S+-*lox*P-*lox*P-P*pgk* with genomic DNA of *A. limacinum* OUC_EG as the template; B: Blank control.

Plasmid p18SCPGC was transformed into *A. limacinum* OUC88, and a transformant, named *A. limacinum* OUC_CG, was selected on solid medium containing chloramphenicol. The *Cm^r^* antibiotic marker gene, integrated into the genome of *A. limacinum* OUC_CG, was detected by PCR using the Cm-F/R primer, and the genomic DNA of *A. limacinum* OUC88, OUC_CG and pACYCDuet-1 plasmid as the templates, respectively. Electrophoresis results (Figure 2a) showed that the amplified fragment had a size of between 500 bp to 750 bp, which is consistent with the length of the *Cm^r^* gene (660 bp). After sequencing, the amplified fragment was exactly the Cm resistance gene, which showed that *A. limacinum* OUC_CG was a positive transformant.

### 2.3. Screening of the Transformant in the Second Electrotransformation Step

Plasmid pSH65 was transformed into cells of *A. limacinum* OUC_CG by electroporation, and transformants were selected on solid medium containing both zeocin and chloramphenicol. Then the transformants were cultured in a liquid galactose induction medium at 23 °C for 48 h and plated onto solid medium containing zeocin. After that, the induced transformants were inoculated onto two solid media containing zeocin and chloramphenicol, respectively. The positive transformants grew normally on the zeocin solid medium, but were unable to grow on the chloramphenicol solid medium, indicating that the *Cm^r^* gene has been deleted from recombinants in the initial step. 

The recombinants were detected by PCR and sequencing using the 18S+-F/P*pgk*-R primer (Table 1). The result showed that the *Cm^r^* gene, located between the two *lox*P sites, had been successfully removed from *A. limacinum* OUC_CG by the action of Cre recombinase (Figure 2b), and further confirmed that the *GAL1* promoter of *S. cerevisiae* on the pSH65 plasmid could play the role of promoting the expression of *Cre* in *A. limacinum*. The desired candidate transformant was sub-cultured in medium without antibiotics and the cultures were spread on two solid media, with and without zeocin, respectively. When the culture could no longer grow on zeocin solid medium, the final recombinant had been obtained and was named *A. limacinum* OUC_EG. 

**Figure 3:**
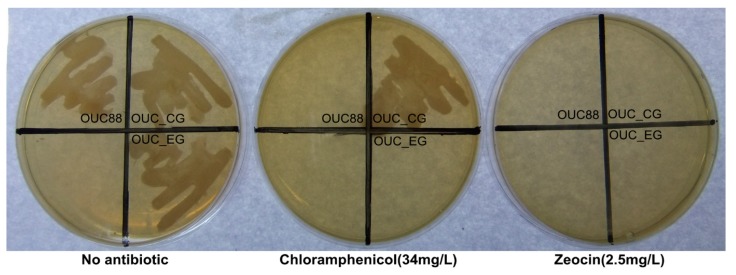
The growth of *A. limacinum* OUC88, *A. limacinum* OUC_CG and *A. limacinum* OUC_EG on different solid media.

The *A. limacinum* OUC88, OUC_CG and OUC_EG strains were inoculated on three solid media, no antibiotics, 100 mg chloramphenicol/L and 5 mg zeocin/L respectively (Figure 3). *A. limacinum* OUC_CG was able to grow on solid medium without antibiotics or on medium with chloramphenicol, but could not grow on solid medium containing zeocin. There were no strains that grew on the medium containing zeocin. *A. limacinum* OUC88 and OUC_EG could grow normally only on medium without antibiotics. The results indicated that the antibiotic resistance genes had been successfully removed from the recombinant *A. limacinum*.

### 2.4. Hybridization Detection in Southern Blotting

The genomic DNA of *A. limacinum* OUC88, OUC_CG and OUC_EG were extracted using a Yeast DNAiso Kit. Through agarose electrophoresis, the genomic DNAs of all the samples were found to be intact and of high purity, and thus could be used in southern blotting.

**Figure 4:**
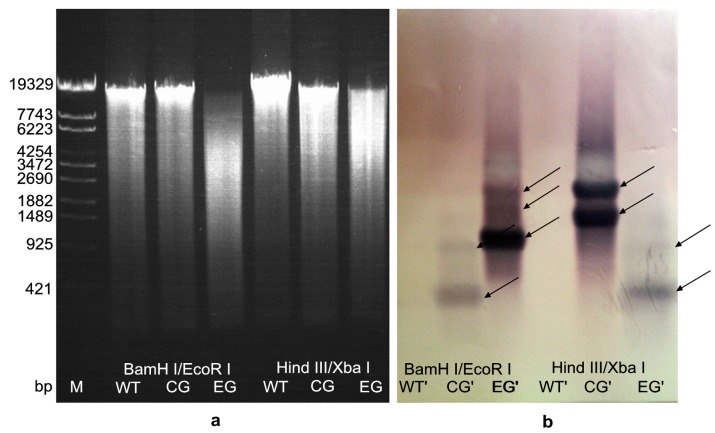
Southern blotting analysis of *egfp* in the genomic DNA of transformants (**a**) The results of total DNA digested by restriction enzymes. Molecular-size of markers (bp) is shown on the left. The genomic DNA of *A. limacinum* OUC88 (WT), *A. limacinum* OUC_CG and *A. limacinum* OUC_EG were double digested with either *Bam*HI/*Eco*RI or *Hin*dIII/*Xba*I; (**b**) The southern-blot hybridization results for the *egfp* gene. The number of fragment copies as deduced from the comparison of the hybridization bands are depicted for the transformants.

Genomic DNAs of all samples were individually digested by *Bam*HI/*Eco*RI and *Xb*aI/*Hin*dIII enzymes respectively, and the digestion fragments were homodispersed by agarose electrophoresis (Figure 4a). The hybridization signals for southern blotting were obtained using *egfp* as the probe (Figure 4b). *A. limacinum* OUC88 had no hybridization signal. For *A. limacinum* OUC_CG and OUC_EG, more than one hybridization bands were found after digestion with either *Bam*HI/*Eco*RI or *Hin*dIII/*Xba*I. These bands represent the presence of *egfp* fragments, thus the results indicate that the *egfp* gene had been integrated into the genome of *A. limacinum* OUC_CG and OUC_EG. The southern blotting results showed that the copy number of *egfp* gene in the genome of *A. limacinum* OUC_CG and OUC_EG was at least one-copy, and further confirmed that the *egfp* in *A. limacinum* OUC_EG was still present after the *Cm^r^* gene had been removed from *A. limacinum* OUC_CG.

### 2.5. Fluorescence Detection of Recombinants

**Figure 5:**
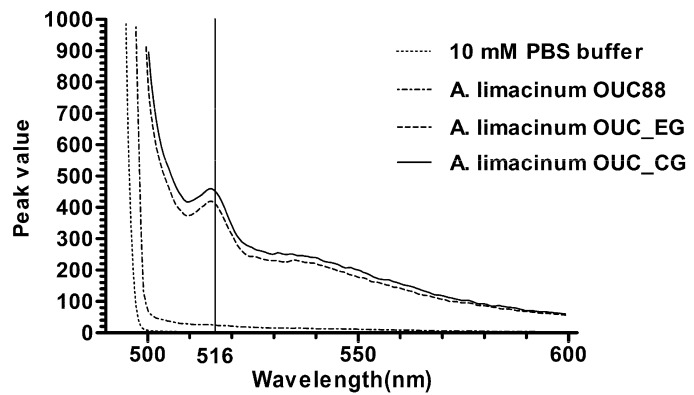
Fluorescence measurements for the recombinants.

With 10 mM PBS buffer as the blank control, and *A. limacinum* OUC88 as the negative control, the fluorescence spectra for *A. limacinum* OUC_CG and OUC_EG were determined. As shown in Figure 5, fluorescence was detected in both *A. limacinum* OUC_CG and OUC_EG, with excitation at 488 nm and an emission peak at 516 nm, which is the characteristic fluorescence of EGFP. Thus, EGFP was expressed in *A. limacinum* OUC_CG and OUC_EG, which proves that the *egfp* gene was integrated into the genome of *A. limacinum* in the first electroporation and its expression was unchanged after the *Cm^r^* gene had been removed from *A. limacinum* OUC_CG in the second electroporation.

**Figure 6:**
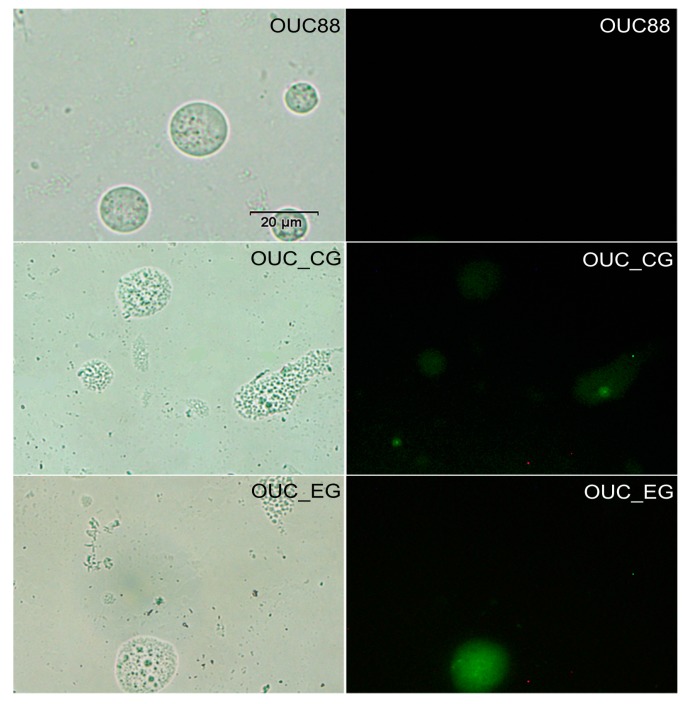
Fluorescence microscopic analysis of the *A. limacinum* transformant (bars = 20 μm).

Finally, the transformants were examined by fluorescence microscopy. The results showed EGFP was expressed stably in *A. limacinum* OUC_CG and OUC_EG, confirming the expression of the *egfp* gene in the transgenic *A. limacinum* (Figure 6).

## 3. Experimental Section

### 3.1. Strains and Media

The *A. limacinum* OUC88 strain was preserved in the China General Microbiological Culture Collection Center (CGMCC No.1240, Beijing, China) and used as the host for the transformation experiment. Solid medium (6% (w/v) glucose, 2% (w/v) yeast extract and 2% (w/v) agar) with a salinity equivalent to 50% that of seawater was used for the conservation and selection of *A.* *limacinum* transformants at 23 °C; Liquid medium (7% (w/v) glucose, 2% (w/v) yeast extract and 2% (w/v) sodium glutamate) with a salinity equivalent to 50% that of seawater was used for the propagation of *A. limacinum* at 23 °C. Liquid galactose induction medium was used to induce the Cre enzyme responsible for removing *Cm^r^* from the transformants. Galactose replaced glucose in this liquid medium.

### 3.2. Plasmids

The plasmids pEGFP-1 and pACYCDuet-1 were purchased from Clontech (Otsu, Japan, Cat.No.6086-1) and Novagen (Billerica, MA, USA, Cat.No.71147-3) respectively; Plasmids pTUAH (Figure 1a) and pSH65 were a gift from Z.M. Chi (Ocean University of China, Qingdao, China.). The pSH65 plasmid contained the sequences of the *Cre* recombinase gene, *GAL1* promoter and *TEF1* promoter of *Saccharomyces cerevisiae*, *CYC1* terminator, *Ble^r^* and *Amp^r^* resistance genes. It was useful to express the Cre recombinase under the control of galactose and markers flanked *lox*P would be removed from the genome of transformant [14,15]. At the same time, the plasmid pSH65 was unstable and would be disappeared in transformants when transformants were subcultured reach over a certain number of times.

The plasmid p18SZPC (Figure 1c) was constructed in our previous work based on plasmid pTEF1/Zeo (V50320, Invitrogen, Carlsbad, CA, USA), which contained sequences of the 18S rDNA of *A. limacinum* (GenBank: HM042908.2), the *PGK* promoter of *S. cerevisiae*, the *CYC1* terminator, and antibiotic resistance genes *Ble^r^* and *Amp^r^*.

### 3.3. Construction of the Plasmid p18SCPGC

The plasmid p18SCPGC was constructed to contain 18S rDNA sequences, *egfp* as a reporter gene, the *Cm^r^* antibiotic resistance gene and two *lox*P sites. The 18S rDNA sequences were used as the homologous recombination sites to integrate the heterologous genes into *A. limacinum*. The *egfp* reporter gene was used to detect the expression of the heterologous gene in *A. limacinum*. The *Cm^r^* gene was used for screening transgenic strains of *A. limacinum*, and was located between the two *lox*P sites. The *Cm^r^* gene was subsequently removed by the inducible expression of Cre recombinase. 

(1) The plasmid pTUAH without *Hyg^r^* was amplified from pTUAH by PCR with primer pair pTUAH-F/R (Table 1). The primer pair Cm-F/R (Table 1) was used to amplify the antibiotic resistance gene, *Cm^r^*, from the vector pACYCDuet-1. The resistance gene *Cm^r^* and the vector pTUAH were ligated to each other and the resulting vector was designated as plasmid pUCMU (Figure 1b).

(2) The coding region of *egfp* was amplified from vector pEGFP-1 with primers EGFP-F/R (Table 1) and introduced into the plasmid p18SZPC between *Bam*HI and *Xba*I, and the resulting vector was named plasmid p18SZPGC (Figure 1d).

(3) The fragment *lox*P-*TEF1*-*EM7*-*Cm^r^*-*CYC1*-*lox*P was obtained from the plasmid pUCMU by PCR with primer pair *lox*P-F/R (Table 1) and ligated into plasmid p18SZPGC between *Eco*RI and *Sac*I. The resulting vector was named plasmid p18SCPGC (Figure 1e).

**Table 1:** Primer pairs used to amplify genes described in this study.

Primers	Sequence (from 5ʹ to 3ʹ)	Template	Product	Length (bp)
Cm-F	ATGGAGAAAAAAATCACTGGAT	Plasmid pACYCDuet-1	*Cm^r^*	660
Cm-R	TTACGCCCCGCCCTGCCACTCA			
pTUAH-F	CACGTCCGACGGCGGCCCGAC	Plasmid pTUAH	pTUAH	4168
pTUAH-R	GGTTTAGTTCCTCACCTTGTCG			
EGFP-F	CGCGGATCCATGGTGAGCAAGGGCGAGGA	Plasmid pEGFP-1	*egfp*	720
EGFP-R	TGCTCTAGATTACTTGTACAGCTCGTCCA			
*lox*P-F	CGAGCTCCTGCTAACATCAAAAGGCCT	Plasmid pUCMU	*lox*P-*Cm*^r^-*lox*P	1536
*lox*P-R	CCGGAATTCATCTTGACTGATTTTTCCATGG			
18S+-F	GCGGGGCCCGTAGTGTACTGGACTACGGTG	Genomic DNA of different transformants	18S+-*lox*P-*Cm^r^*-*lox*P-P*pgk*	3655
P*pgk*-R	CGCGGATCCATATTTGTTGTAAAAAGTAGATAATTAC		18S+-*lox*P-*lox*P-P*pgk*	2185

Note: The underlined portions of the primer sequence show the restriction enzyme cutting sites.

### 3.4. Electrotransformation

The circular plasmid was transformed into *A. limacinum* by electrotransformation following the procedure described by Cheng [2]. The cells of *A. limacinum* OUC88 were cultured overnight in 10 mL liquid medium, inoculated into 50 mL fresh medium and grown to a logarithmic phase (OD_600_ ≈ 1.5). Cells were harvested by centrifugation at 8000× *g* for 5 min at 4 °C. The resuspended pellet was washed sequentially with 10 mL ice-cold sterile water and 10 mL of 1 M sorbitol solution, and then diluted to 10^6^ cells/mL with 1 M sorbitol solution. The *A. limacinum* competent cells were mixed with circular plasmid DNA and transferred to a 0.2-cm cuvette for electroporation. The most suitable electroporation parameters were: 1.8 kV/cm, 200 Ω and 50 μF. After electroporation, the solution was recovered in 1 mL liquid medium and incubated at 28 °C, 200 rpm/min, for 1 h.

### 3.5. The Screening of Transformants

*A. limacinum* was not sensitive to ampicillin, kanamycin or streptomycin, but sensitive to chloramphenicol and zeocin [16]. Even a high concentration of the former group of antibiotics (50–300 mg/L) could not inhibit the growth of *A. limacinum* and its survival rate was still over 80%. However, the survival rate of *A. limacinum* cultured with chloramphenicol was below 13% at a concentration of 25.5–68 mg/L, and only 2.10%–2.79% at a zeocin concentration of 2.5–4.0 mg/L. Accordingly, 100 mg chloramphenicol/l and 5 mg zeocin/L were used respectively for screening of transformants after the two transformation steps in this research.

In the first step, plasmid p18SCPGC was transformed into *A. limacinum* OUC88, which integrated the *lox*P-*Cm^r^*-*lox*P-*egfp* fragment into the genome by homologous recombination with the 18S rDNA sequences of *A. limacinum* OUC88. The recombinants were screened on solid medium containing 100 mg chloramphenicol/L, and were confirmed by PCR amplification of the *Cm^r^* gene. After sequencing of the amplified *Cm^r^* fragment, the recombinant was used in the following step.

In the second step, plasmid pSH65 was transformed into the previous recombinant. After selection on solid medium with zeocin and incubation at 23 °C for 48 h in the dark, the transformants were cultured in liquid galactose induction medium in the dark (200 rpm, 23 °C, 48 h) to induce the expression of Cre recombinase in plasmid pSH65 so as to excise the *Cm^r^* gene located between the *lox*P sites. Subsequently, the transformants were inoculated simultaneously on two solid media containing chloramphenicol and zeocin, respectively. One reombinant that could grow on zeocin solid medium, but could not grow on chloramphenicol solid medium, was selected as a candidate recombinant. This candidate recombinant was tested by PCR, using the primers Cm-F/R to confirm the deletion of the *Cm^r^* gene.

Finally, the candidate recombinant was sub-cultured in medium without antibiotics for 10 generations during which plasmid pSH65 could be lost [17]. The cultures were spread on two solid media with and without zeocin, respectively. When the culture could no longer grow on zeocin solid medium, the final recombinant had been obtained.

### 3.6. Southern Blotting of the Recombinants

Genomic DNA was extracted from *A. limacinum* OUC88 and the transformants respectively using a Yeast DNAiso Kit (D9082, Takara, Otsu, Japan). DNA samples were digested with two groups of restriction enzymes (*Bam*HI/*Eco*RI, *Xba*I/*Hin*dIII) and for each sample, the resultant DNA fragments were separated on an 0.8% agarose gel and transferred to a nitrocellulose membrane (0.22 μm, Pall, New York, NY, USA) by siphon blotting. The DNA fragment for *egfp*, used as the probe, was prepared by PCR using the primers EGFP-F/R. Subsequently, the purified fragments were labeled with DIG before use as DNA hybridization probes. Probe detection in the Southern blotting was performed using a DIG High Prime DNA Labeling and Detection Starter Kit I (for color detection with NBT/BCIP) (Cat.NO.11745832910, Roche, Basel, Switzerland).

### 3.7. Fluorescence Detection of the Recombinants

The *A. limacinum* OUC88 and transformants were cultured overnight in the liquid medium, inoculated into fresh medium and grown to logarithmic phase. Cells were harvested by centrifugation at 8000× *g* for 5 min at 4 °C. The collected cells were washed twice with sterile water and re-suspended in 10 mM PBS buffer (pH 7.2). Cell suspensions of *A. limacinum* were diluted to 10^6^ cells/mL with 10 mM PBS buffer and the fluorescence spectrum was measured using an F4500 fluorescence spectrophotometer (Hitachi, Tokyo, Japan). Detection parameters for measuring the fluorescence spectrum were as follows: scanning speed 1200 nm/min, slit width 5.0 nm, voltage 950 V and excitation wavelength 488 nm.

## 4. Conclusions

*A. limacinum* is an ideal industrial species for producing DHA [18,19,20,21,22], and its application prospects are very broad [23,24,25]. Optimization of culture conditions and breeding of mutants have been common techniques to improve the yield of DHA in *A. limacinum*; However, these methods cannot not improve the content of DHA readily or in a controlled manner. At present, genetic engineering has become an effective method to change genetically-based biological traits. The greatest advantage of genetic engineering is that it is highly specific, and use of this technology can avoid problems, such as the uncertain nature of mutations and the work-load of screening in traditional mutation breeding. In *A. limacinum,* the methods for genetic transformation have already been established. Electrotransformation and *Agrobacterium* *tumefaciens*-mediated transformation have been successfully applied to *A. limacinum*. Homologous recombination also has been shown to be effective in integrating a foreign gene into the genomic DNA of *A. limacinum*. In order to make the transformants suitable for industrial application, the selectable antibiotic resistance marker genes should be removed. Accordingly, the Cre/*lox*P system has been applied to *A. limacinum* for the first time in this research.

First, two *lox*P loci, the chloramphenicol resistance gene and the *egfp* gene were integrated into the genome of *A. limacinum* OUC88, using the 18S rDNA sequences of *A. limacinum* as the homologous recombination sites. Verified by antibiotic resistance screening, PCR and sequencing, a transformant named *A. limacinum* OUC_CG was obtained. Next, the plasmid pSH65 was incorporated into *A. limacinum* OUC_CG and Cre recombinase was expressed by galactose induction. After antibiotic resistance screening, *A. limacinum* OUC_EG was found to grow on the solid medium containing 5 mg zeocin/L, but not on the solid medium having 100 mg chloramphenicol/L. As proven by PCR, the *Cm^r^* sequence located between the two *lox*P loci had been successfully removed from the genome of *A. limacinum* OUC_EG. During continuous sub-culturing on medium without antibiotics, the recombinant *A. limacinum* OUC_EG lost the plasmid pSH65 and completely lost its antibiotic resistance. Subsequently, through southern blotting, *egfp* was proven to have integrated into the genome of *A. limacinum* with a copy number of at least one. By fluorescence detection of EGFP, *A. limacinum* OUC88 had no emission peak, whereas *A. limacinum* OUC_CG and OUC_EG both had an obvious emission peak at 516 nm after excitation at 488 nm. These observations suggest that EGFP was expressed in *A. limacinum* OUC_CG and OUC_EG, and that the presence of *egfp* was not affected by the deletion of the *Cm^r^* gene by the action of Cre recombinase.

In this paper, the Cre/*lox*P site-specific recombination system was introduced into *A. limacinum* OUC88. Using the 18S rDNA sequences as the homologous recombination sites, *egfp* was integrated into the genome of *A. limacinum* and expressed successfully. In addition, the antibiotic resistance gene was eliminated from *A. limacinum*. This research has laid an experimental foundation for using genetic engineering technology to modify the traits of *A. limacinum* and obtain transformed strains that have no antibiotic resistance marker, which make them potentially useful for applications in industry.
